# Evaluation of a local government “shelter and van” intervention to improve safety and reduce alcohol-related harm

**DOI:** 10.1186/s12889-018-6245-4

**Published:** 2018-12-12

**Authors:** Bernadette M. Ward, Belinda O’Sullivan, Penny Buykx

**Affiliations:** 1 School of Rural Health, Monash University, PO Box 666, Bendigo, VIC 3552 Australia; 2 School of Health and Related Research, University of Sheffield, Sheffield, UK

**Keywords:** Evaluation, Alcohol-related harm, Local government, Community intervention, Entertainment precinct

## Abstract

**Background:**

The entertainment precincts of cities, while contributing to local economies, need to be carefully managed to mitigate harms. Individual behaviours and government regulation have typically been the foci of interventions aimed at reducing alcohol-related harm. Little is known about how changes to the built environment might influence alcohol-related harms in these settings. The aim of this study was to explore how a public shelter and a volunteer-funded and staffed mobile van in a regional city influenced perceptions of safety and reduction in alcohol-related harm.

**Methods:**

An intrinsic case-study approach was used. Document reviews, qualitative interviews with 16 key informants (volunteers, licensees, police, local business owners, patrons, community members and security guards), observation, and secondary data analysis were conducted in 2016. A conceptual framework of the causative pathways linking the drivers of alcohol consumption with social and health outcomes was used to inform the analysis.

**Results:**

The shelter and van were frequently utilised but there was no significant association with a reduction in the proportion of alcohol-related hospital emergency department presentations or police incident reports. Occupational health and safety risks were identified for the volunteers which had no management plan.

**Conclusions:**

The findings highlight the challenge faced by local governments/authorities wanting to provide community-based interventions to complement other evidence-based approaches to reduce alcohol-related harm. Local governments/authorities with restricted regulatory oversight need to collaborate with key agencies for targeted upstream and evidence-based alcohol prevention and management interventions before investing resources. Such approaches are critical for improving community safety as well as health and social outcomes in communities at greatest risk of alcohol-related harm.

## Background

Globally, alcohol consumption is the leading risk factor for premature death and disability amongst people aged 15–49 years [1]. While public health efforts to reduce the misuse of alcohol are multifaceted, there is robust evidence that policy can have a positive effect on reducing alcohol-related harm [2]. The population-based interventions with the strongest evidence for effectiveness are limiting alcohol availability, reducing drink-driving, and increasing the price of alcohol [2, 3]. The implementation of policies such as alcohol taxation is often at a national government level but local governments/authorities also have a role as they are commonly called up to respond to concerns about alcohol-related harms in their jurisdiction; particularly in entertainment districts where ease of access to multiple licensed venues is associated with increased alcohol-related harms [4].

Internationally, the function of local governments/authorities is very broad and may including economic development, planning and monitoring, public health, the development and enforcement of law, advocacy and service delivery [5, 6]. To achieve a balance between health and non-health sector goals, local governments are at times required to foster collaborative efforts between internal departments and with external partner agencies [7].

Action by local governments/authorities is partly determined by the breadth of their portfolio and available resources. In England and Wales, local government authorities are responsible for liquor licensing and trading arrangements [8]. A number of local authorities with high outlet density and high rates of alcohol-related harm have implemented cumulative impact policies (CIPs) which assume, in a defined geographical area, a licence application will be refused unless the applicant can demonstrate they do not compromise alcohol-related harm licensing objectives [9]. These have been associated with a reduction in alcohol-related hospital admissions [10]. In contrast, in Australia, local governments have a relatively minor role in liquor licensing. Responsibility rests with state/territory governments, who also have a role in ensuring that liquor outlets conform to local planning zone restrictions [11].

In Australia, local governments/authorities actively promote their role in creating physical environments that contribute to community health and wellbeing [12]. They often invest in projects aimed at reducing alcohol-related harm [13].The cost impact of alcohol-related harms is substantial; it is estimated that for every dollar (AUD$) spent on prevention (e.g. community wellbeing and public health staffing), $2.78 is spent on responding to the impacts of alcohol (e.g. cleaning, security) and an additional $0.43 cents on operational costs (e.g. licensing application assessments) [14]. Some Australian local governments have implemented a range of community interventions that appear to have good face validity but are not supported by empirical evidence [15, 16]. For example, time-series analyses found the introduction of close-circuit television (CCTV) cameras, a Night-Watch Radio Program, Liquor Accords and patron bans, in four metropolitan/rural Australian cities, were not significantly associated with a change in alcohol-related emergency department (ED) injury presentations [15, 16]. The findings of recent systematic reviews suggest restrictions on late night trading hours are effective in reducing alcohol-related violence but it is not known whether the same is true for ‘lockouts’ [17, 18].

Individual behaviours and government regulation have typically been the foci of interventions aimed at reducing alcohol-related harm. In recent years there has also been an increased emphasis on the effect of drinking practices (e.g. where and when alcohol consumption occurs) [19] and the role of the built environment on health behaviours [20] suggesting neighbourhood infrastructure is associated with drinking practices [21]. For example, in the United States mixed model analyses adjusted for individual behavioural, socio-economic and demographic confounders, found that men who have positive *perceptions* of neighbourhood safety (e.g. sidewalk availability and maintenance, proximity and affordability of facilities, local crime rates and traffic) were eight times less likely to binge drink alcohol [21].

In response to reports of alcohol-related police reports [22] and socially inappropriate behaviours [23], a local government in a rural city in south-eastern Australia introduced two related built-environment interventions aimed at improving public safety by reducing alcohol-related harm. Firstly, in 2011, a volunteer-staffed, mobile van began operating in the central business district (CBD) entertainment precinct on Saturday nights. The van, located adjacent to the central-city taxi rank operates from 10 pm to 4 am on Saturday nights. Up to four volunteer staff offer social and crisis (e.g. first aid, referral to health services) support, health promotion materials (e.g. condoms, water), and transport assistance to community members and patrons of licensed premises. Secondly, in 2013, the local government constructed a secure sheltered area with lighting and amenities (toilet, seating) adjacent to the taxi rank along with a structured taxi queuing system [23, 24]. On Saturday nights both facilities were supervised by security guards sub-contracted to the local government authority. The skills of these staff were similar to those of security guards (‘bouncers’) employed by venues to monitor safety within and immediately surrounding venues, but their remit was the shelter and mobile van area. The local government had previously implemented a range of interventions aimed at reducing alcohol-related harm including a liquor accord, closed camera television (CCTV) cameras, ‘lockouts’ (whereby patrons cannot enter premises after 2 am), ‘no-shots’, ‘patron ban’, ‘polycarbonate drinkware’, a night-rider bus and a taxi rank [25, 26] (See Table 1).

**Table 1:** Local government interventions 1997–2013 aimed at reducing alcohol-related harm

Name of Intervention	Date implemented	Description
Liquor Accord	1997 (February)	Aimed to ensure and maintain ethical conduct within all licensed premises. At the time, Licensees declared their commitment to the promotion of the responsible service of alcohol (RSA).
Surveillance cameras	1998	Introduced in the entertainment precinct [22].
Review of Liquor Accord	2002	This resulted in 114 licensees signing a commitment to implement the Accord [22].
2 am venue lockout	2007	No person is allowed to enter a licensed premise after 2 am [26, 53].
Night-rider bus	2007 (July)	To pick up clients from venues on demand. This intervention was co-funded by licensees, police and local government. The bus was discontinued after three years (June 2010) because of inconsistent contributions from licensees [54, 55].
Patron Ban	No date	Licensees can ban a patron from their venue for troublesome behaviour, and police can also ban someone from a venue.
No shots policy	2010	“No shots after 2 am” policy [25, 26].
Polycarbonate drinkware	No date	Implemented in all 5 am venues.
Taxi ranks	2004	Three taxi ranks were in operation with varying levels of demand.
Supervised taxi ranks	October 2005	A supervised taxi rank [23]. Two security guards supervise the rank from 2 am–6 am on Sunday mornings [23].
Chill Out Van	February 2011	A van that is staffed by volunteers to provide a range of services.
Safe Transport Shelter	February 2013	Architecturally designed shelter with amenities. Supervised taxi rank moved to this location.

The aim of this study was to examine the utilisation of the secure sheltered area and the volunteer-staffed mobile van and explore how this change in the built environment related to perceptions of increased safety and reduced alcohol-related harm in the CBD entertainment precinct in the period 2010–2015.

Earlier research based in similar sized rural Australian cities suggests community-based interventions are not associated with significant reductions in emergency department (ED) presentations or assault rates [16] but little is known about how changes to the built environment might influence alcohol-related harm in night-time economies.

## Methods

**Fig. 1:**
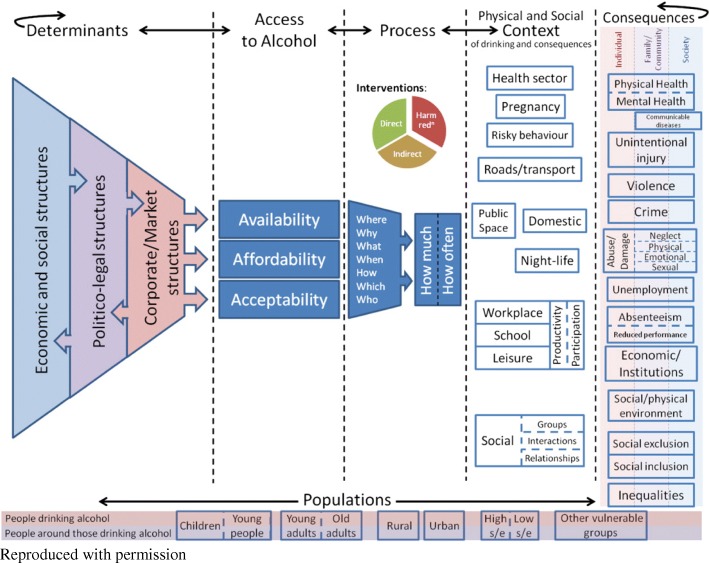
Conceptual framework of the causative pathways linking proximal drivers of alcohol consumption with distal health and social outcomes

An intrinsic case study approach was used to provide in-depth understanding of the interventions via the analysis of multiple types of data [27]. A conceptual framework of the causative pathways linking the drivers of alcohol consumption with social and health outcomes was used to inform the interpretation of the findings [3] (see Fig. 1).

### Setting

**Fig. 2:**
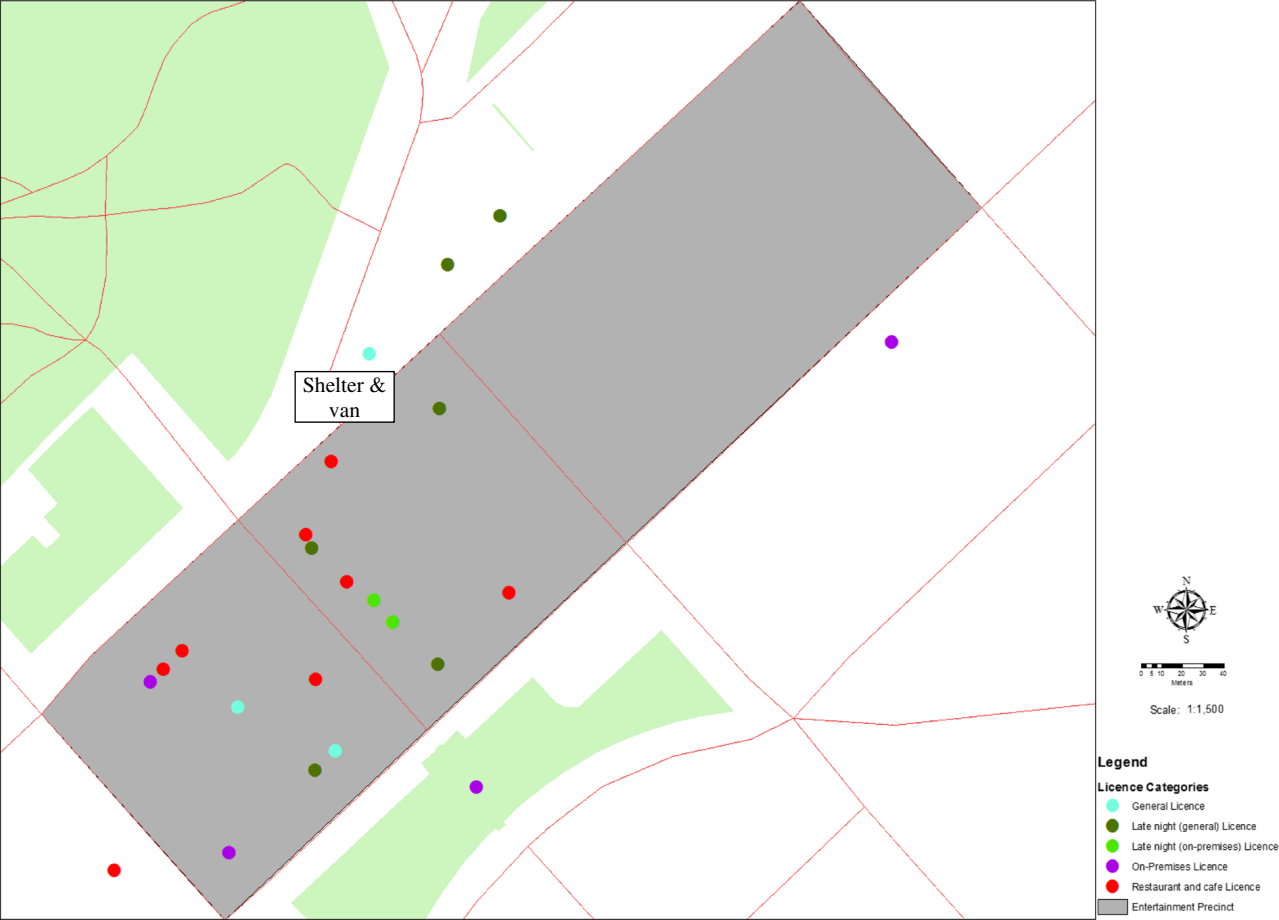
Licenced Venues by licence type in the entertainment precinct

The local government area population is approximately 110,000 people, covers 3000 km^2^ and is 150 km north of Melbourne [28]. The CBD entertainment precinct is seven urban blocks and at the time of the study included 25 licensed premises, seventeen of which closed at or before 1 am and eight after 1 am [29]. Of the eight operating after 1 am, two had ‘Late’ night (on-premises) licences, and six had late night (general) licences (i.e. able to supply take-away alcohol). The geographical distribution of these venues is presented in Fig. 2. The proportion of the population that utilise the precinct was unknown, but collectively, the premises had capacity for more than 3000 patrons per night.

### Data sources and collection

The case study included primary and secondary data. Primary data was collected via qualitative semi-structured key informant (KI) interviews, a face-to-face quantitative venue patron survey, and observations of the shelter and mobile van. Interviews with nearby business operators, volunteers, security guards and other non-government stakeholders explored their awareness and perceptions of the interventions and their perceived effects on reducing alcohol-related harm. The street-intercept survey (with space for additional comments via free text), of venue patrons who were using the shelter and/or van was conducted between 11 pm and 4 am on two Friday and Saturday nights. The questions were based on those used in an earlier Australian study [30] Close-ended questions focused on patrons’ sociodemographic characteristics, awareness, perceptions of safety and utilisation of the facilities (e.g. *Does the presence of the shelter (van) increase your confidence about the safety of being out in the CBD at night? Do you think this area has decreased the negative effects of alcohol (yes/no) near the pubs and clubs?*) with options to provide comments. Qualitative observational data were collected by three on-site researchers on Friday/Saturday/Sunday nights. Researchers noted the use of the amenities, types of anti-social behaviour, activities of and interactions between intervention security guards, venue security guards and volunteers.

Secondary data collected included police reports of non-domestic assaults, property damage and other anti-social behaviours in the entertainment precinct (2010–2015), local hospital ED (located within 1 km of the shelter) presentations for simple intoxications of alcohol, security guard incident reports (July 2012–July 2014) and shelter CCTV footage. Licensed premises’ private Facebook groups were joined to assist with the qualitative assessment of responsible promotion and service of alcohol.

### Analysis

The qualitative data was managed in NVivo 10 [31] and quantitative data in SPSS 22 [32].

#### Qualitative analysis

Qualitative KI interview transcripts, security guard observation reports, field notes, observation data, and Facebook data were explored using thematic analysis [33]. The interview data were collected and analysed concurrently to explore emerging themes in subsequent interviews. For legal reasons we are unable to provide direct quotes from interviews with police officers, though their data was incorporated via thematic analysis.

#### Quantitative analysis

Quantitative patron survey, police, ED, security guard incident reporting, and CCTV footage data were analysed using descriptive statistics. Aggregated police reports from the entertainment precinct during 2010–2015 were coded and analysed. Police incident time and dates was coded to reflect high, medium and low alcohol hours as follows: high-alcohol hours - Friday and Saturday nights between 8.00 pm and 6.00 am; medium-alcohol hours - Sunday through Thursday nights between 8.00 pm and 6.00 am; low-alcohol hours - all days between 6.00 am and 8.00 pm [34]. Aggregated ED data of uniquely identified “simple intoxication” reports were examined by year, age, gender and time of arrival in ED and where possible, matched with high, medium and low alcohol hours [34]. Security guard reports were aggregated by time, month and year and where possible, matched with high, medium and low alcohol hours. The analyses were triangulated to enhance the validity of the results.

Ethics approval was provided by Monash University Human Research Ethics Committee and Victoria Police.

## Results

Primary data included 26 h of observation, a survey of 74 patrons (32 women [mean age 26 years], 31 men [mean age 22 years], missing data *n* = 11), interviews with 16 KIs and three hours of Facebook data collection. The qualitative and quantitative results were incorporated and presented by key themes.

### Intoxicated patrons

Several KIs spoke about the “drunk” and “drug affected” people in and around the shelter. For example:

*People are so drunk they don’t know which way is up and they are a danger to themselves from falling and hitting their heads, walking on the road and falling in front of traffic, or from being robbed or beaten up. They are often literally “legless”*. (volunteer)

Observation and KIs data suggested that many of these were coming out of licensed premises. This suggests non-responsible service of alcohol and was consistent with non-responsible promotion of alcohol via the Facebook groups of several licensed premises. Despite this, one licensee reported:

*The worst possible thing any staff member can do in this business and it’s literally a fireable offence on the spot is to serve an intoxicated patron. I literally am the invisible policeman I think now they* [the police] *know the clubs are doing a pretty good job at curtailing that kind of behaviour and they’re giving us a little bit more leeway.*

### The role and utilisation of the shelter and van

More than half (40/74) of patrons reported they perceived it was safe to wait for a taxi and/or meet with friends at the shelter and mobile van and, when clean, use the toilets. This was confirmed by observational data. KIs reported the volunteers at the mobile van were highly regarded. Patrons and volunteers confirmed our observations of the use of the mobile van to access support, information, breathalysing kits, refreshments, warm blankets, flat shoes and a portable gas heater surrounded by outdoor chairs. Volunteers reported walking around to check for intoxicated people in alleys and backstreets, “taking people home in their own car”, and “informally triaging” people for service referral (e.g. ambulance, ED, police) and:

*We help people reconnect with their friends, from whom they have become separated, help them if they have lost their phones or wallets. We sometimes phone someone to come and pick them up.*

Crowds of people were regularly observed walking between licenced premises, and through the shelter. This was confirmed by a report from a licensee who suggested there were large numbers of people in, and around, the licenced premises who also used the shelter and the van:

*On a super super super busy peak night, you could expect that we could have at least anywhere up to seventeen hundred and fifty people walking past our front door making their way from club to club, from club to taxis, club to the park, clubs to home.*

### Perceptions of safety

Forty-two percent (31/74) of patrons thought having the shelter and mobile van reduced harm from alcohol. This was consistent with KI interview data analysis. Nonetheless, 71% of patrons reported the shelter improved their confidence about the safety of being in the CBD at night. A number of patrons reported feeling “safe at night anyway”. There were specific concerns about the design of the shelter. These included inadequate screening of the toilets:

*Sometimes men just stand at the bowl to urinate or zip up as the walk out. Sometime people urinate in the outside hand basin.* (Restaurant owner)

the design of the seating:

*The concrete seating, with metal inserts that stick up, is dangerous as intoxicated people have been known to fall on them. When a person’s head hits the seat sometimes serious injuries occur. This can also happen during fights which are quite frequent.* (Restaurant owner)

and the design of the roof:

*I understand why it’s so tall because it stops people from climbing up on top of it, which is a good thing but at the same time when it rains it doesn’t stop the rain or the wind from people sheltering there.* (Patron)

The design of the shelter was thought to compromise safety of the taxi rank. It was the intention of the architects that a lighting system embedded in the pavement would indicate how the taxi queue should be formed (i.e. curving around the concrete seats) but there is no signage to explain this. The taxi queue was so confusing that it could promote conflicts between patrons in queues. The security guard said:

*People don’t know where to stand so they sometimes stand in the wrong place because a fight ensues* [sic] *between those who think someone is queue jumping and someone else who thinks he/she is in the right line.*

Many key informants and surveyed patrons commented on the hygiene of the toilets, and this was consistent with our observations. Cleanliness of the toilets deteriorated during the evening/night with toilet paper scattered about and vomit, urine and faeces in the cubicles. This was also a problem for local business owners who had to hose down the area outside their business every Sunday and Monday morning.

### Harms and related security activity

Key informants reported harms included personal injury, anti-social behaviour, and more serious incidents including assaults in and around the area in which the shelter and mobile van were located. The security guards acted to contain and prevent anti-social behaviour and manage the taxi queue. Security guard incident reports were infrequent (approximately once a month) and somewhat unpredictable: the types of incidents ranged from minor altercations such as jumping the taxi queue, to more severe altercations involving violence (e.g. glassing, punching, wrestling, slapping and threatening behaviour). Guards frequently de-escalated violence and occasionally reported assaults. However, their well-intentioned efforts to reduce problems sometimes inadvertently resulted in escalation. For example, prioritising people in the taxi queue sometimes resulted in a fight. Volunteers at the van reported aggression related to high demand for limited taxis:

*Security [at the rank] put a group into the maxi cab, and 3 people got upset and started threatening security saying ‘they were going to kill them’. They settled down when security made them realise there was another cab for them.*

The taxi association interviewee reported taxi drivers are not allowed to refuse drunk people except for people who have “messed themselves” (i.e. urinated, defecated, vomited). He indicated it can be difficult to get night drivers because of the “aggravation” involved. Some drivers had been “traumatised” by incidents on Saturday nights, and so have given up night work. Despite this he suggested that the situation had improved in recent years:

*There’s a lot of mutual respect with security, and the drivers understand that without the security guys there, it would be a nightmare because they’ve had it before when the space* [shelter] *was less organised.*

The taxi rank security guards had limited interaction with the volunteers and venue security guards. As a result, the venue security staff called upon the volunteers to provide security. For example:

*Sometimes we’ve been outnumbered and it is terrifying, …and instead of getting any assistance from the guards it was the ladies who work the van who came over to help try and talk some sense into the guys we were trying to separate. The rage settles a bit when they see it’s a woman the ladies use some pretty good methods in order to settle the situation down and I'm very impressed but at the same time I was even more terrified that some idiot was going to clock one of the ladies, that was my concern.* (Licensee)

Implicit in this statement was the idea that licensees may have assumed that the local government security staff were a resource funded to work right up to the night club door. The point at which police should be called was not clear. Sometimes police were not called until volunteers were already in danger. For example:

*If there’s a punch up with people and the guards and the volunteers are all involved and our guys* [licensed premises security guards] *are involved then I’ll call the cops because I don't want to see the volunteers getting it, but half the time the cops are completely stretched so thin that it could be anywhere between twelve and twenty minutes before a* [police] *van will rock up.* (Licensee)

The willingness of licensees to use their own security guards and volunteers, instead of police meant venues avoided a formal police incident report. For example:

*Primarily because we don’t want to involve them in nightclub instances because ninety-nine percent of the time we’re completely innocent in that incident and we get written up with what’s called a licensed premises incident report [LPIR]. If anything should happen in the future whereby we were to be called in front of any type of tribunal to say whether or not I'm fit to hold a liquor license the first thing the prosecution does is open up, oh look at this, we’ve got twenty-five LPIRs from this venue in the last ten years, it’s obviously a hot spot for violence and anti-social behaviour.* (Licensee)

Consistent with taxi rank security staff reports and our observations, the volunteers reported working with other services at the site such as police, ambulance officers, security guards and licensees; going into the club if requested. For example:

*We have a system where they flash torches if the security guard [at the venues] needs help. Then they do the same for us. We can flash our torches and they respond. The security guards are very good but their role is limited to the area around the taxi rank and they are not permitted to do anything outside that area.* (Volunteer)

The licensee supported having the volunteers available more frequently. “I’d have them two nights a week. If it was available, I think it would be great to do it.”

**Fig. 3:**
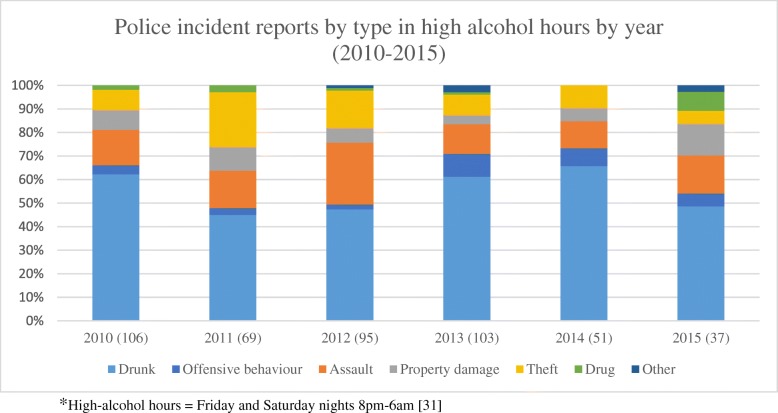
Proportion of police incident reports, by type, in high-alcohol hours* and year (2010–2015)

Police data revealed few incidents. This was consistent with non-police interviewee reports and our observations of minimal police attendance. The document analysis found qualitative reports by police that they are increasing surveillance in and around the entertainment precinct [35]. However, there was a lack of quantitative evidence supporting this. Between 2010 and 2015 the annual average number of police incident reports from the entertainment precinct was 195 but the numbers fluctuated by year (see Fig. 3). Across the years, in any hours, the most common incident was “drunk” followed by “theft”. While there was an increase in the proportion of offensive behaviour incident reports, since 2012 the trends across incident types during high alcohol hours were inconsistent. More than half (56%) of all “drunk” incidents occurred in high-alcohol hours. Similarly, 45% of assaults and 37% of offensive behaviour incident reports occurred in these hours. Drunk and assault incidents made up the highest proportion of all incidents in high-alcohol hours.

The analysis of the 2015 CCTV found 20 incidents occurring in the immediate area of the shelter; that is, on average, one incident every 2–3 weeks. These included fights and anti-social behaviour. However, no time of day data was available so it was not possible to determine the association between alcohol hours, these incidents and police reports.

### Hospital presentations for alcohol intoxication

As per Table 2, from 2010 to 2013 there were 264 ED presentations for simple intoxication of alcohol; 51% were aged 15–24 years and 52% were male. Twenty-two percent presented on a weekday between 12.01 am and 7.59 am, 12% on a Saturday in the same time frame and 23% on Sundays between 12.01 am and 11.59 am. Peak months were January (*n* = 45), April (*n* = 40), and October (*n* = 41). While there was no consistent pattern in ED presentations across years or by Saturday/Sunday mornings the proportion of young adults aged 15–24 years presenting with simple intoxication remained constant.

**Table 2:** Proportion of ED simple alcohol intoxications (excludes poisoning) by year (2010–2013) and their age group, gender and day/time of week (%)

Year	n (%)	15–24 yrs. %	Female %	Mon-Fri 12.01–7.59 am %	Sat 12.01–7.59 am %	Sun 12.01–11:59 am %	Mon-Sat 12.01–7.59 am & Sun – 11.59 am %
All years	264						
2010–11	72 (18)	50	51	38	14	10	62
2011–12	94 (23)	49	50	16	15	23	54
2012–13	98 (23)	46	48	22	10	27	59

## Discussion

The aim of this study was to explore the relationship between the purpose built shelter and co-located volunteer-staffed mobile van and perceptions of safety and alcohol-related harm in a rural city CBD entertainment precinct between 2010 and 2015. No consistent association was evident between the interventions and changes in alcohol-related offences and behaviours, as reflected in police incident reports and alcohol-related ED presentations.

In terms of reducing entertainment precinct alcohol-related harms, most of the positive aspects of these interventions relate to the physical and social context of drinking. Their purpose is to address the consequences (e.g. unintentional injury, violence, crime) of exposure to the major risk factor; high-risk alcohol consumption. That is not to say these activities are not beneficial. Instead, it suggests that, in this setting, it may also be beneficial to target interventions towards *access to alcohol* and patterns of high-risk drinking. It was evident that the intention to address the context of drinking increased reliance on volunteers for evacuating patrons and the management of taxi queues stimulated aggression by some patrons.

The conceptual framework by Martineau et al., (2013) (Fig. 1) highlights the role of economic, policy and market factors in access to alcohol and associated potential consequences of alcohol misuse. Economic development including entertainment venues is commonly a priority for local governments/authorities; particularly in rural cities where unemployment rates are typically higher than those seen in metropolitan areas [36]. However, in rural cities such as the one described in this study, patrons’ drinking and the observed levels of alcohol-related harm may be higher than those seen in metropolitan settings [37, 38]. This places additional burden on rural local governments to respond appropriately to public concerns about the misuse of alcohol and associated harms. Many rural cities face the additional impact of ease of access to multiple proximal venues, which has been associated with increased alcohol-related harm [39].

In most communities, local police have a responsibility have a role in ensuring licensees are not breaching their liquor licensing conditions. The contradiction between the very low levels of police incident reports for drunkenness and the drunkenness reported by KIs in this study suggests there may be grounds for additional resources to enable police to increase their monitoring of the licensed premises to ensure licensees were meeting their obligations; to not admit and/or serve intoxicated or underage patrons. The intoxication level observed suggests that licensees were not adhering to their responsible service of alcohol obligations. The use of volunteers to assist with venue security appeared inappropriate; it is the role of the licensee and where necessary, the police.

Public safety is an important priority for local governments/authorities [12, 40]. We found a strong sense of good will and respect for the security guards at the shelter and the volunteers in the mobile van and these enabled access to social support and sheltered amenities. Given the dynamic nature of patrons and other consumers entering the area during high-risk alcohol hours, it seems that good marshalling of people, and the co-operation between the security guards and volunteers plays a role in safety within the entertainment precinct. However, the role of, and risk for, local governments/authorities in providing a ‘safe’ place for staff and volunteers needs further exploration and should be addressed within an occupational health and safety framework. We found the scope of practice for staff and volunteers managing anti-social behaviour was poorly defined. In high-risk situations (such as volunteers walking laneways, using personal transport, attending or entering licensed premises to manage intoxicated patrons), the safety of personnel is compromised. Role clarification and training (particularly for volunteers) is needed to enhance personal safety of those working in and around these facilities.

Many of the most effective interventions to reduce entertainment precinct alcohol-related harm are within the power of higher (i.e. not local) levels of Australian governments; for example limiting alcohol availability (including reducing outlet density), drink-driving programs and increasing the price of alcohol [2–4]. There is mixed evidence for community-level and alcohol service setting interventions [3, 10, 16] but some of these are more effective when accompanied by other strategies. These include lockout policies [41, 42]; mandatory server and management compliance with responsible service of alcohol [3, 43]; increased police patrols [44] and mandatory ID scanners that are linked to police or other regulatory agency monitoring activities [15, 45]. There is little evidence to support awareness campaigns, night-watch radio programs [3] or on-premises patron education programs [46]. Simulation modelling suggest 24 h access to public transport [47] may reduce alcohol-related harm in metropolitan entertainment precincts but the feasibility and generalisability of this to rural cities is unknown. A night-rider bus in the study setting was not financially sustainable and subsequently discontinued. In locations where there is a shortage of taxis and no other public transport, efforts to reduce the number of patrons (sometimes intoxicated) congregated in one place while waiting for a taxi should be considered.

In Australia, unlike the United Kingdom, local governments have a proximal role in scrutinising and informing the approval process of licensing applications submitted to the state liquor commission (albeit limited) and, in turn, monitoring the density of liquor outlets in their local government area. Local governments/authorities without authority over licensing and trading hours are limited in their ability to implement effective evidence-based strategies that are specifically targeted at reducing alcohol-related harm [3]. Despite this, many continue to invest large amounts of funds in alcohol-harm reduction interventions; often in response to public demand [48]. While there is some evidence that the built environment can effect drinking behaviour [21], this is not specific to entertainment precincts. The implementation and building of the permanent shelter described in this study cost more than one million Australian dollars and was supported by state and local government and private businesses [49, 50] and by definition, was not subject to a trial. Since its construction, nearby liquor outlets have closed while others have opened outside of this geographically defined entertainment district [51]. The permanent nature of the shelter limits the ability of the local government to adapt their response to a potential change in the geographical foci of liquor outlets in the CBD.

Not all factors in the physical and social context are the responsibility of local government (e.g. roads/transport, health, workplace), and this is intrinsically linked to the roles of agencies in other sectors in and outside of local government. In addition, much of the alcohol-related harm and subsequent resource allocation is linked to high-risk drinking in licensed premises. Licensees and mobile van volunteers interviewed in this study had different understandings of the role of the local government funded security guards. In the absence of liquor licensing decision making authority, further assessments and discussions are needed to determine the role of Australian local government in providing resource intensive distal supports (e.g. cleaning, security services) for high-risk alcohol consumption at commercial licensed premises. Many local governments/authorities prioritise a safe, vibrant entertainment precinct as a part of building liveable cities. However, there are costs associated with this and the Victorian 2012 amendment to the Local Government Act 1989 meant that local government can no longer apply differential rating to late night licensed venues [52]. In light of this, we suggest authorities consider the financial/non-financial resources they are willing to commit to the night-time economy and subsidisation of high-risk alcohol consumption as part of planning their entertainment precincts.

There were limitations to this study. While we attempted to take into account a range of data sources, we were limited to using a range of secondary administrative data with potential inconsistencies of periods of observation and restricted variables. Ideally, evaluations should be planned in advance as part of an intervention to ensure adequate data infrastructure is more structured to answer the questions posed. This is particularly so where interventions are cumulative, and stepped evaluation approaches may be needed. In addition, the lack of access to weekly geographical focus and “operational time” of the CCTVs and the resultant raw CCTV data meant that its usefulness was limited. Similarly, relatively ‘old’ taxi queue waiting period data and the absence of time and day information on police postcode data suggest that some of the analyses should be interpreted with caution. While we provided an in-depth description of one local government approach to reducing alcohol-related harm, we did not have information about other cases. By definition, rural cities are unique in their geography and characteristics and so the generalisability of the results may be limited. Notwithstanding these limitations our findings highlight the importance of transport infrastructure and occupational health and safety support for staff and volunteers at such facilities.

## Conclusions

The results suggest that the volunteer-staffed van and co-located shelter were valued and frequently utilised. However, there is little evidence that this infrastructure reduced alcohol-related harm in this rural city CBD entertainment precinct. The findings highlight the challenge faced by local governments/authorities wanting to provide community-based interventions to complement other evidence-based approaches (e.g. liquor licensing restrictions) to reduce alcohol-related harm. Local governments/authorities with restricted regulatory oversight, need to collaborate with key agencies for targeted upstream and evidence-based alcohol prevention and management interventions before investing resources in aspects of volunteer services and the built environment. Such approaches are critical for improving community safety as well as health and social outcomes in communities at greatest risk of alcohol-related harm.

## Abbreviations

CBD: Central business district
CCTV: Close-circuit television
CIP: Cumulative Impact Policies
CoGB: City of Greater Bendigo
ED: Emergency Department
KI: Key informants
LPIR: Licensed premises incident report

## References

[1] Griswold MG, Fullman N, Hawley C, Arian N, Zimsen SRM, Tymeson HD, Venkateswaran V, Tapp AD, Forouzanfar MH, Salama JS (2018). Alcohol use and burden for 195 countries and territories, 1990-2016: a systematic analysis for the global burden of disease study 2016. Lancet.

[2] Babor T, Caetano R, Casswell S, Edwards G, Giesbrecht N, Graham K, Grube JW, Hill L, Holder H, Homel R (2010). Alcohol: no ordinary commodity: research and public policy.

[3] Martineau F, Tyner E, Lorenc T, Petticrew M, Lock K (2013). Population-level interventions to reduce alcohol-related harm: an overview of systematic reviews. Prev Med.

[4] Livingston M (2011). Alcohol outlet density and harm: comparing the impacts on violence and chronic harms. Drug and Alcohol Review.

[5] Victorian Local Governance Association, What is local government?, Melbourne, 2016. http://www.goodgovernance.org.au/about-good-governance/what-is-local-government/. Accessed 29 Nov 2018.

[6] United Cities and Local Governments, The global network of cities**,** Local and Regional Governments**,** Spain. 2014. https://www.uclg.org/. Accessed 29 Nov 2018.

[7] World Health Organization. Health in all policies - Helsinki statement framework for country action in. Geneva: World Health Organization; 2014. http://www.ngos4healthpromotion.net/wordpressa4hp/wp-content/uploads/2016/11/helsinki.pdf. Accessed 29 Nov 2018.

[8] Her Majesty's Stationary Office. Licensing act 2003. https://www.legislation.gov.uk/ukpga/2003/17/contents. Accessed 29 Nov 2018.

[9] de Vocht F, Heron J, Campbell R, Egan M, Mooney JD, Angus C, Brennan A, Hickman M. Testing the impact of local alcohol licencing policies on reported crime rates in England. J Epidemiol Community Health. 2016. 10.1136/jech-2016-207753. 10.1136/jech-2016-207753 PMC5284476 27514936

[10] de Vocht F, Heron J, Angus C, Brennan A, Mooney J, Lock K, Campbell R, Hickman M. Measurable effects of local alcohol licensing policies on population health in England. J Epidemiol Community Health. 2015. 10.1136/jech-2015-206040. 10.1136/jech-2015-206040 PMC4789824 26555369

[11] Victorian Government. Liquor control reform act. 1998. https://www.vcglr.vic.gov.au/liquor/restaurant-cafe/understand-your-liquor-licence/legislation-and-regulations. Accessed 29 Nov 2018.

[12] Australian Local Government Association (ALGA). Strategic Priorities 2014–17. Canberra; 2016. https://alga.asn.au. Accessed 29 Nov 2018.

[13] ALGA. ALGA News June Canberra. 2010. http://alga.asn.au/newsletter/newsletters.ALGA.NEWS.20100621. Accessed 29 Nov 2018.

[14] ALGA, Is it Your Shout Again?: Uncovering Councils' Hidden Alcohol Costs. Canberra. 2010.http://councilreferendum.com.au/newsletter/newsletters.ALGA.NEWS.20100910. Accessed 29 Nov 2018.

[15] Miller P, Sonderlund A, Coomber K, Palmer D, Gillham K, Tindall J, Wiggers J (2011). Do community interventions targeting licensed venues reduce alcohol-related emergency department presentations?. Drug Alcohol Rev.

[16] Curtis A, Coomber K, Droste N, Hyder S, Palmer D, Miller PG (2017). Effectiveness of community-based interventions for reducing alcohol-related harm in two metropolitan and two regional sites in Victoria, Australia. Drug and Alcohol Review.

[17] Taylor N, Miller P, Coomber K, Mayshak R, Zahnow R, Patafio B, Burn M, Ferris J. A mapping review of evaluations of alcohol policy restrictions targeting alcohol-related harm in night-time entertainment precincts. Int J Drug Policy. 2018;62:1–13 10.1016/j.drugpo.2018.09.012. Accessed 29 Nov 2018. 10.1016/j.drugpo.2018.09.012 30347331

[18] Wilkinson C, Livingston M, Room R (2016). Impacts of changes to trading hours of liquor licences on alcohol-related harm: a systematic review 2005–2015. Public Health Res Pract.

[19] Meier PS, Alan W, John H (2018). All drinking is not equal: how a social practice theory lens could enhance public health research on alcohol and other health behaviours. Addiction.

[20] Berke EM, Vernez-Moudon A (2014). Built environment change: a framework to support health-enhancing behaviour through environmental policy and health research. J Epidemiol Community Health.

[21] Jitnarin N, Heinrich K, Haddock C, Hughey J, Berkel L, Poston W (2015). Neighborhood environment perceptions and the likelihood of smoking and alcohol use. Int J Environ Res Public Health.

[22] Entertainment Precinct Working Group, Key Findings. CoGB, Bendigo. 2004. https://www.bendigo.vic.gov.au/. Accessed 29 Nov 2018.

[23] CoGB. Regional Development Victoria Expression of Interest Form. 2011. https://www.bendigo.vic.gov.au/.. Accessed 29 Nov 2018.

[24] CoGB (2011). Minutes Safe Transport Space Meeting, 6 July.

[25] Shots banned as Accord cracks down on liquor in Bendigo, Bendigo Advertiser. Fairfax Regional Media. 11 February 2010. https://www.bendigoadvertiser.com.au/story/707244/shots-banned-as-accord-cracks-down-on-liquor-louts-in-bendigo/. Accessed 29 Nov 2018.

[26] Eade R, Breen K. City of Greater Bendigo Safe Transport Space: Report of Independent Panel. Bendigo.: CoGB; 2010. https://www.bendigo.vic.gov.au/. Accessed 29 Nov 2018.

[27] Creswell J (2003). Research design: qualitative, Quantiative and mixed methods approaches, 2nd edition edn.

[28] Australian Bureau of Statistics (ABS), *Census Quick Stats* 2012, Canberra 2011. http://www.censusdata.abs.gov.au/census_services/getproduct/census/2011/quickstat/202?opendocument&navpos=220. Accessed 29 Nov 2018.

[29] Victorian Commission for Gambling and Liquor Regulation, Victorian liquor licences by location. 2016. https://www.vcglr.vic.gov.au/i-want/find-venue-data. Accessed 29 Nov 2018.

[30] Miller P, Tindall J, Sonderlund A, Groombridge D, Lecathelinais C, Gillham K, McFarlane L, Fd G, Droste N, Palmer D, et al. Dealing with Alcohol and the Night-Time Economy (DANTE): Monograph no. 43. Canberra: National Drug Law Enforcement Research Fund; 2012. http://www.ndlerf.gov.au/publications/monographs/monograph-43. Accessed 29 Nov 2018.

[31] QSR International. NVivo qualitative data analysis software: Version 10, QSR International Pty Ltd; 2012.

[32] SPSS Inc. SPSS for Windows Version 17.0. Chicago; 2008.

[33] Liamputtong P, Ezzy D. Qualitative research methods: South Melbourne Oxford University Press; 2005.

[34] Dietze P, McElwee P, Heale P, Jonas H, Hanlin K, Cvetkovski S. The Victorian alcohol statistics handbook volume 2: alcohol-related serious road injury and assault in Victoria, 1992–1999: Melbourne Turning Point Alcohol and Drug Centre Inc; 2001. www.turningpoint.org.au. Accessed 29 Nov 2018.

[35] CoGB, 2013, Community safety strategy 2013–2016, September, Bendigo. https://www.bendigo.vic.gov.au/. Accessed 29 Nov 2018.

[36] National Rural Health Alliance Inc. Income inequality experienced by the people of rural and remote Australia: Submission to the Senate Inquiry into the Extent of Income Inequality in Australia, Canberra. 2014. http://ruralhealth.org.au/sites/default/files/documents/nrha-policy-document/submissions/sub-income-inequality-inquiry-15-oct-2014.pdf

[37] Roxburgh A, Miller P, Dunn M (2013). Patterns of alcohol, tobacco and cannabis use and related harm in city, regional and remote areas of Australia. Int J Drug Policy.

[38] Curtis A, Coomber K, Droste N, Hyder S, Mayshak R, Lam T, Gilmore W, Chikritzhs T, Miller PG. Consumption plans for the rest of the night among Australian nightlife patrons. 2017;**6**(1):7. 10.7895/ijadr.v6i1.243.

[39] Campbell CA, Hahn RA, Elder R, Brewer R, Chattopadhyay S, Fielding J, Naimi TS, Toomey T, Lawrence B, Middleton JC (2009). The effectiveness of limiting alcohol outlet density as a means of reducing excessive alcohol consumption and alcohol-related harms. Am J Prev Med.

[40] Public Health England, *Public Health in Local Government*, Department of Health, London. https://assets.publishing.service.gov.uk/government/uploads/system/uploads/attachment_data/file/216708/dh_131904.pdf. Accessed 29 Nov 2018.

[41] de Andrade D, Homel R, Townsley M: Trouble in paradise: The crime and health outcomes of the Surfers Paradise licensed venue lockout. Drug Alcohol Rev 2016:n/a-n/a. 10.1111/dar.12384 10.1111/dar.12384 26913775

[42] Miller P, Palmer D, McFarlane E, Curtis A (2014). Key stakeholder views of venue lockouts in Newcastle and Geelong. Crime Prevention & Community Safety.

[43] Scherer M, Fell JC, Thomas S, Voas RB (2015). Effects of dram shop, responsible beverage service training, and state alcohol control Laws on underage drinking driver fatal crash ratios. Traffic Inj Prev.

[44] Goss CW, Van Bramer LD, Gliner JA, Porter TR, Roberts IG, DiGuiseppi C. Increased police patrols for preventing alcohol-impaired driving**.** Cochrane Database Syst Rev 2008, Issue 4. Art. No.: CD005242. doi: 10.1002/14651858.CD005242.pub2. 10.1002/14651858.CD005242.pub2 PMC13160403 18843684

[45] Miller P, Curtis A, Palmer D, Busija L, Tindall J, Droste N, Gillham K, Coomber K, Wiggers J (2014). Changes in injury-related hospital emergency department presentations associated with the imposition of regulatory versus voluntary licensing conditions on licensed venues in two cities. Drug Alcohol Rev.

[46] Ker, K; Chinnock, P (2006) Interventions in the alcohol server setting for preventing injuries. Cochrane Database Syst Rev, 2 (2). CD005244. ISSN 1469-493X 10.1002/14651858.CD005244.pub2. 10.1002/14651858.CD005244.pub2 16625630

[47] Scott N, Hart A, Wilson J, Livingston M, Moore D, Dietze P (2016). The effects of extended public transport operating hours and venue lockout policies on drinking-related harms in Melbourne, Australia: results from SimDrink, an agent-based simulation model. Int J Drug Policy.

[48] Sartori, E. Bendigo safe space to be launched, The Bendigo Advertiser. Fairfax Regional Media. 22 February, 2011. https://www.bendigoadvertiser.com.au/story/714655/bendigo-safe-space-to-be-launched/. Accessed 29 Nov 2018.

[49] Premier of Victoria, Safe Transport Space boost for Bendigo community, Media release. Government of Victoria Melbourne. 2013. https://www.premier.vic.gov.au/category/media-release. Accessed 29 Nov 2018.

[50] Firm backs chill space, The Bendigo Weekly, p. 9. Fairfax Regional Media. 16 June, 2011. https://issuu.com/bgoweekly/docs/716jpg. Accessed 29 Nov 2018.

[51] Victorian Commission for Gambling and Liquor Regulation. Victorian liquor licences by location. 2018. https://www.vcglr.vic.gov.au/i-want/find-venue-data. Accessed 29 Nov 2018.

[52] Department of Planning and Community Development. Ministerial guidelines on differential rates. Melbourne: Victorian Government; 2013. https://www.localgovernment.vic.gov.au/__data/assets/pdf_file/0022/74821/Ministerial_Guidelines_for_Differential_Rating_April_2013-PDF.pdf. Accessed 29 Nov 2018.

[53] Lockout official -- no nightclub entry after 2am**.** Bendigo Advertiser. 28 August, 2007. https://www.bendigoadvertiser.com.au/story/682524/lockout-official-no-nightclub-entry-after-2am. Accessed 29 Nov 2018.

[54] Nightrider bus set to continue**.** Bendigo Advertiser, Fairfax Regional Media. 4 March, 2010. https://www.bendigoadvertiser.com.au/story/708262/nightrider-bus-set-to-continue/. Accessed 29 Nov 2018.

[55] No more Night Rider, Bendigo Advertiser, Fairfax Regional Media. 2010. https://www.bendigoadvertiser.com.au/story/709638/no-more-night-rider/. Accessed 29 Nov 2018.

